# Variability in plasma rifampicin concentrations and role of *SLCO1B1*, *ABCB1*, *AADAC2* and *CES2* genotypes in Ethiopian patients with tuberculosis

**DOI:** 10.1080/23744235.2024.2309348

**Published:** 2024-02-05

**Authors:** Tesemma Sileshi, Eyasu Makonnen, Nigus Fikrie Telele, Victoria Barclay, Alimuddin Zumla, Eleni Aklillu

**Affiliations:** a Department of Pharmacy, Ambo University, Ambo, Ethiopia;; b Department of Pharmacology and Clinical Pharmacy, Addis Ababa University, Addis Ababa, Ethiopia;; c Center for Innovative Drug Development and Therapeutic Trials for Africa (CDT-Africa), Addis Ababa University, Addis Ababa, Ethiopia;; d Department of Laboratory Medicines, Karolinska Institutet, Stockholm, Sweden;; e Department of Infection, Division of Infection and Immunity, University College London; NIHR Biomedical Research Centre, UCL Hospitals NHS Foundation Trust, London, UK;; f Department of Global Public Health, Karolinska Institutet, Karolinska University Hospital, Stockholm, Sweden

**Keywords:** Rifampicin, pharmacokinetics, pharmacogenetics, *SLCO1B1*, *ABCB1*, *AADAC*, *CES-*2, genotype, Ethiopia, tuberculosis

## Abstract

**Background:**

Rifampicin, a key drug against tuberculosis (TB), displays wide between-patient pharmacokinetics variability and concentration-dependent antimicrobial effect. We investigated variability in plasma rifampicin concentrations and the role of *SLCO1B1*, *ABCB1*, arylacetamide deacetylase (*AADAC*) and carboxylesterase 2 (*CES-2*) genotypes in Ethiopian patients with TB.

**Methods:**

We enrolled adult patients with newly diagnosed TB (*n* = 119) who had received 2 weeks of rifampicin-based anti-TB therapy. Venous blood samples were obtained at three time points post-dose. Genotypes for *SLCO1B1* (*c.388A>G*, *c.521T>C*), *ABCB1* (*c.3435C>T*, c.4036A>G), *AADACc.841G>A* and *CES-2* (*c.269-965A>G*) were determined. Rifampicin plasma concentration was quantified using LC-MS/MS. Predictors of rifampicin *C*_max_ and AUC_0–7h_ were analysed.

**Results:**

The median rifampicin *C*_max_ and AUC_0–7_ were 6.76 μg/mL (IQR 5.37–8.48) and 17.05 μg·h/mL (IQR 13.87–22.26), respectively. Only 30.3% of patients achieved the therapeutic efficacy threshold (*C*_max_>8 μg/mL). The allele frequency for SLCO1B1* 1B (*c.388A>G*), SLCO1B1* 5 (*c.521T>C*), *ABCB1 c.3435C>T*, *ABCB1c.4036A>G*, *AADAC c.841G>A* and *CES-2 c.269-965A>G* were 2.2%, 20.2%, 24.4%, 14.6%, 86.1% and 30.6%, respectively. Sex, rifampicin dose and *ABCB1c.4036A>G*, genotypes were significant predictors of rifampicin *C*_max_ and AUC_0–7_. *AADACc.841G>A* genotypes were significant predictors of rifampicin *C*_max_. There was no significant influence of *SLCO1B1* (*c.388A>G,* c*.521T>C*), *ABCB1c.3435C>T* and *CES-2 c.269-965A>G* on rifampicin plasma exposure variability.

**Conclusions:**

Subtherapeutic rifampicin plasma concentrations occurred in two-thirds of Ethiopian TB patients. Rifampicin exposure varied with sex, dose and genotypes. *AADACc.841G/G* and *ABCB1c.4036A/A* genotypes and male patients are at higher risk of lower rifampicin plasma exposure. The impact on TB treatment outcomes and whether high-dose rifampicin is required to improve therapeutic efficacy requires further investigation.

## Introduction

Whilst effective tuberculosis (TB) treatment has been available for the past seven decades, the latest 2022 WHO Annual Global Tuberculosis Report highlights that TB remains a leading cause of death from an infectious disease worldwide [1]. Considerable success have been achieved in treatment outcomes since the introduction of rifampicin in 1970. However, the global increase in HIV incidence, poor adherence to 6-month therapy and suboptimal drug concentrations due to interindividual pharmacokinetic variations of first-line antitubercular drugs have contributed to the emergence of resistance to TB drugs [2–4]. Drug-resistant TB is a concern in East African countries [5]. Ethiopia is among the top 30 countries with the highest TB and TB-HIV burden with an incidence of 119 cases per 100,000 people in 2021 [1].

A combination of rifampicin with isoniazid is the backbone of modern anti-TB therapy. Rifampicin has concentration-dependent bactericidal activity [6]. The microbial killing of rifampicin was linked to the ratio of the area under the concentration-time curve and the minimum inhibitory concentration (AUC/MIC) and the maximum concentration (*C*_max_)/MIC (*C*_max_/MIC) ratio. Rifampicin prevents resistance to itself and attains sufficient bactericidal effect at a free *C*_max_/MIC ratio of ≥175 [7,8]. A rifampicin *C*_max_ between 8 and 24 μg/mL is considered optimal and *C*_max_ below 4 μg/mL is a risk factor for treatment failure [9].

Rifampicin undergoes hepatic metabolism by genetically polymorphic carboxylesterases (CES) and arylacetamide deacetylase (AADAC), a serine esterase to 25-deacetylrifampicin [10]. Rifampicin pharmacokinetics and treatment outcomes display wide between-patient variations [11,12]. Genetic variation in enzymes and transporter proteins relevant to rifampicin disposition may influence the variability of plasma rifampicin exposure. Previous studies in various populations investigated the impact of genetic variation in *AADAC* and *CES* on rifampicin plasma exposure with varying results [13–16]. Rifampicin is a substrate and inducer of the organic anion transporter polypeptide 1B1 (OAT1B1) encoded by the *SLCO1B1* gene [17] and P-glycoprotein (P-gp) encoded by the *ABCB1* gene [18]. OAT1B1 mediates hepatocellular uptake of rifampicin while P-gp mediates drug efflux. Both *SLCO1B1* and *ABCB1* genes are genetically polymorphic displaying wide between-population variation in enzyme activity and variant allele frequency distributions. In the few published studies investigating the effect of the *SLCO1B1* and *ABCB1* gene polymorphism on rifampicin pharmacokinetics, the result is inconclusive [16,19–22].

The pharmacokinetics and pharmacogenetics of rifampicin display wide between-race and between-population variations, highlighting the need for investigation in different geographic locations where the burden of TB is high. The effect of pharmacogenetic variability in rifampicin pharmacokinetics using a targeted candidate gene approach has been explored in various Asian and Caucasian populations [22–24], but data from sub-Saharan Africa remain scarce. Ethiopia is the seventh top high-TB burden country globally [1]and the second most populous nation in Africa. The pharmacogenetics of *SLCO1B1* and *ABCB1* in Ethiopians differs from that of other black African populations and inhabitants of European origin [23,25,26]. In this study, we examined the variability in rifampicin *C*_max_ and AUC_0–7_ in Ethiopian TB patients in relation to the recommended target concentration for optimal therapeutic efficacy and the impact of common functional genetic variants in *SLCO1B1* (rs2306283 and rs4149056), *ABCB1* (rs1045642 and rs3842), *CES 2* (rs4783745) and *AADAC* (rs1803155) on between-patient variability in rifampicin plasma concentration.

## Methods

### Study participants and settings

The study participants were newly diagnosed adults aged 18–65 years with either pulmonary or extrapulmonary drug-sensitive *Mycobacterium tuberculosis* attending TB clinics in Addis Ababa (Beletshachew, Teklehymanote, Kazanchis, Woreda 2 and Areda Health Centre). The study was conducted from October 2019 to November 2021.

### Blood sample collection

Blood samples were obtained 2 weeks after treatment initiation during the intensive phase of TB therapy. Following overnight fasting, participants received drugs under direct observation in the morning. A total of 351 venous blood samples were collected in EDTA tubes, with three samples taken at different times from 113 subjects and two times from 6 subjects. The blood sampling points ranged from 1 to 7 h post-dose, with the majority of subjects sampled at 1, 2, 4, or 2, 4, or 6 h post-dose. Plasma was separated immediately and stored at −80 °C at the Department of Pharmacology and Clinical Pharmacy, Addis Ababa University until transported to Karolinska Institutet, Stockholm, Sweden for analysis.

### Ethical approval

Ethical approval was obtained from the Institutional Review Board of the College of Health Sciences at Addis Ababa University and the National Research Ethics Review Committee. All patients were informed about the purpose of the study and those willing to participate and who provided written informed consent were enrolled. The study was conducted following the ethical principle of the Helsinki Declaration.

### Treatment

Study participants received a standard daily dose of rifampicin in combination with isoniazid, pyrazinamide and ethambutol according to the Ethiopian treatment guidelines [27]. Patients with a body weight above 55kg received four fixed-dose combinations (FDC) tablets daily. Patients with a body weight between 40 and 55 kg received three FDC tablets daily and those below 40 kg received two FDC tablets daily. Each FDC tablet contains 150, 75, 400 and 275 mg of rifampicin, isoniazid, pyrazinamide and ethambutol, respectively. Treatment was given as directly observed therapy at a primary health care facility in Addis Ababa, Ethiopia.

### DNA extraction and genotyping

Genomic DNA was extracted from whole blood samples using the QIAmp DNA Blood Midi Kit (QIAGEN GmbH, Hilden, Germany) following the manufacturer’s instructions. Common functional variant alleles in the black African population relevant to rifampicin disposition were selected for genotyping. Genotyping was performed using TaqMan^®^ drug metabolism assay reagents for allelic discrimination (Applied Biosystems Genotyping Assays) as described previously [28] with the following ID numbers: C___8911003_1 for *AADAC2* (c.841G>A, rs1803155), C__31760486_10 for *CES2* (c.269-965A>G, rs4783745), C___7586657_20 for *ABCB1* (3435 C>T, rs1045642), C__11711730_20 for *ABCB1* (c.193A>G, rs3842), C___1901697_20 for *SLCO1B1* (c.388A>G, rs2306283) and C__30633906_10 for *SLCO1B1* (c.521T>C, rs4149056).

The final volume for each reaction was 10 μL, consisting of 1 μL genomic DNA and 9 μL of TaqMan^®^ fast advanced master mix (Applied Biosystems, Waltham, MA, United States), DNA/RNA free water, TaqMan 40X for *SLCO1B1*, *ABCB1* and TaqMan 20 × for *AADAC2* and *CES2* drug metabolism genotyping assays mix (Applied Biosystems). Genotyping was performed by real-time qPCR (Applied Biosystems) equipped with 7500 software V2.3 (Life Technologies Corporation) for allelic discrimination.

### Quantification of rifampicin plasma concentrations

To determine rifampicin plasma concentrations, blood samples were collected 2 weeks after treatment initiation during the intensive phase of TB therapy. After overnight fasting, study participants received drugs under direct observation in the morning. Venous blood was taken in EDTA tubes at three time points from 1 to 7 h post-dose. Plasma was separated immediately and stored at −80° C at the Department of Pharmacology and Clinical Pharmacy, Addis Ababa University until transported to Karolinska Institutet, Stockholm, Sweden for analysis.

Rifampicin plasma concentrations were determined using a liquid chromatography-tandem mass spectrometry (LC-MS/MS) as described previously [11]. The method was validated according to the European Medicines Agency guidelines [29]. The LC-MS/MS system consisted of an Acquity Ultra Performance LC-system coupled to a Xevo TQ-S Micro (Waters, Milford, MA, USA) and aYMC-ultraHT hydrosphere C18, 2 μm, 100 × 2 mm, reversed-phase column (Waters) was used. Sample preparation consisted of protein precipitation with acetonitrile containing deuterated rifampicin as an internal standard. In brief, 100 μL plasma samples were diluted with a 300 μL solution containing the internal standards dissolved in acetonitrile. After shaking for 30 s and 5 min of centrifugation, 150 μL of the supernatant was transferred to another plate. The supernatant dried for 30 min at 35° C and the dried sample was re-suspended with 15 μL methanol and 275 μL 0.1% formic acid for injection. The mobile phase gradient of 0.1% formic acid in Milli-Q pure water, 100% methanol:methanol/Milli-Q pure water:formic acid (10:90:0.1), methanol:Milli-Q pure water:isopropanol:formic acid (70:20:10:0.1), methanol:Milli-Q pure water (10:90). Rifampicin concentrations were calculated by linear regression from a six-point calibration curve. The limits of the quantitation range for rifampicin were 0.1 and 40 μg/mL.

### Pharmacokinetic and statistical analyses

Study participants’ sociodemographic and baseline clinical parameters are summarised as the median and interquartile range (IQR) or as frequency and percentages. The rifampicin *C*_max_ was determined from the available plasma concentrations. The highest concentration observed was taken as *C*_max_. AUC_0–7 h_ calculation was performed using the trapezoidal rule. GraphPad Prism was used to calculate AUC_0–7 h_.

The Shapiro–Wilk test was used to determine the normality of pharmacokinetics data. Non-normally distributed data are presented as median (IQR) and normally distributed as mean (standard deviations [*SD*]). The chi-square test was used to assess correlations between the observed and expected genotype frequencies according to the Hardy–Weinberg equilibrium. All plasma concentration data were log 10 transformed before conducting statistical analyses [29]. The association of each genotype with between-patient variability in *C*_max_ and AUC_0–7_ was analyzed using a one-way analysis of variance, comparing the geometric mean of log-transformed concentration data [30]. Predictors of *C*_max_ and AUC_0–7 h_ of rifampicin were subjected to further analysis through univariate followed by multivariate regression analysis, incorporating study participant characteristics and genotypes as potential predictors. Variables with *p* value <0.2 from the univariate analysis were included in the multivariate regression analysis. Data were analyzed using SPSS version 25 and a *p* value ≤0.05 was considered to indicate statistical significance.

## Results

### Study participants characteristics

Of the 119 study participants, consisting of 62 males and 57 females, 78 were diagnosed with pulmonary TB and 41 had extrapulmonary TB. The median body weight was 54.8 kg (IQR, 48.0–61.7), and the median age was 28 years (IQR, 22 – 35). The mean dose of rifampicin was 9.39 mg/kg (*SD* = 0.98). The prevalence of cigarette, khat and alcohol use was 13.4%, 18.5% and 16.8%, respectively. Notably, a lower percentage of patients with extrapulmonary TB reported cigarette, khat and alcohol use compared to those with pulmonary TB. Furthermore, patients with extrapulmonary TB showed higher rifampicin *C*_max_ (*p* = 0.07) and AUC_0–7_ (*p* = 0.23) values but the differences were not statistically significant. The sociodemographic characteristics of the participants are presented in Table 1.

### Genotype and allele frequency

Study participants were genotyped for *SLCO1B1c.388A>G*, *SLCO1B1 c.521T>C*, *ABCB1 c.3435C>T*, *ABCB1 c.4036A>G*, *AADAC c.841G>A* and *CES-2 c.269-965A>G*. The observed genotype and allele frequency distributions among patients are shown in Table 2. There were no significant differences between observed and expected genotype frequencies according to Hardy–Weinberg equilibrium. The variant allele *SLCO1B1c.388A>G* was frequent (62.2%), while the defective variant allele *SLCO1B1c.521T>C* (*5) was less frequent (20.2%). The minor variant allele frequency for *ABCB1 c.3435T* and *ABCB1 c.4036G* were 24, 4%, and 14.6%, respectively. The variant *AADAC c.841A* variant allele had a much higher frequency (86.1%), whereas the CES-2 c.269–965G allele occurred in 30.6%.

### Rifampicin pharmacokinetics

There was high between-patient variability in rifampicin *C*_max_ (range 1.90–18.57 μg/mL) and AUC_0–7_ (range 3.61–47.1 μg × h/mL). The median rifampicin *C*_max_ was 6.76 μg/mL (IQR 5.33–8.49). Only 30.3% (*n* = 36) of participants achieved the target plasma concentration (> 8 μg/mL) for optimal therapeutic efficacy [31]. *C*_max_ <4 μg/mL, which is associated with risk for treatment failure, was observed in 5 (4.2%) patients. The median AUC_0–7 h_ was 17.1 μg × h/mL (IQR 13.9–22.3).

### Effect of genotype on rifampicin pharmacokinetics

A comparison of the median and geometric mean of rifampicin *C*_max_ and AUC_0–7 h_ between the different genotypes using one-way analysis of variance is presented in Table 3. Although no significant influence of SLCO1B1*1B and SLCO1B1*5 genotype on variation in rifampicin *C*_max_ and AUC_0–7h_ was found, patients homozygous for SLCO1B*5/*5 (*C/C*) had a *C*_max_ below the target concentration. No significant difference in *C*_max_ and AUC_0–7_ was observed in *ABCB1 c.3435C>T* and CES 2 c.269-965A>G genotype groups.

**Figure 1.**
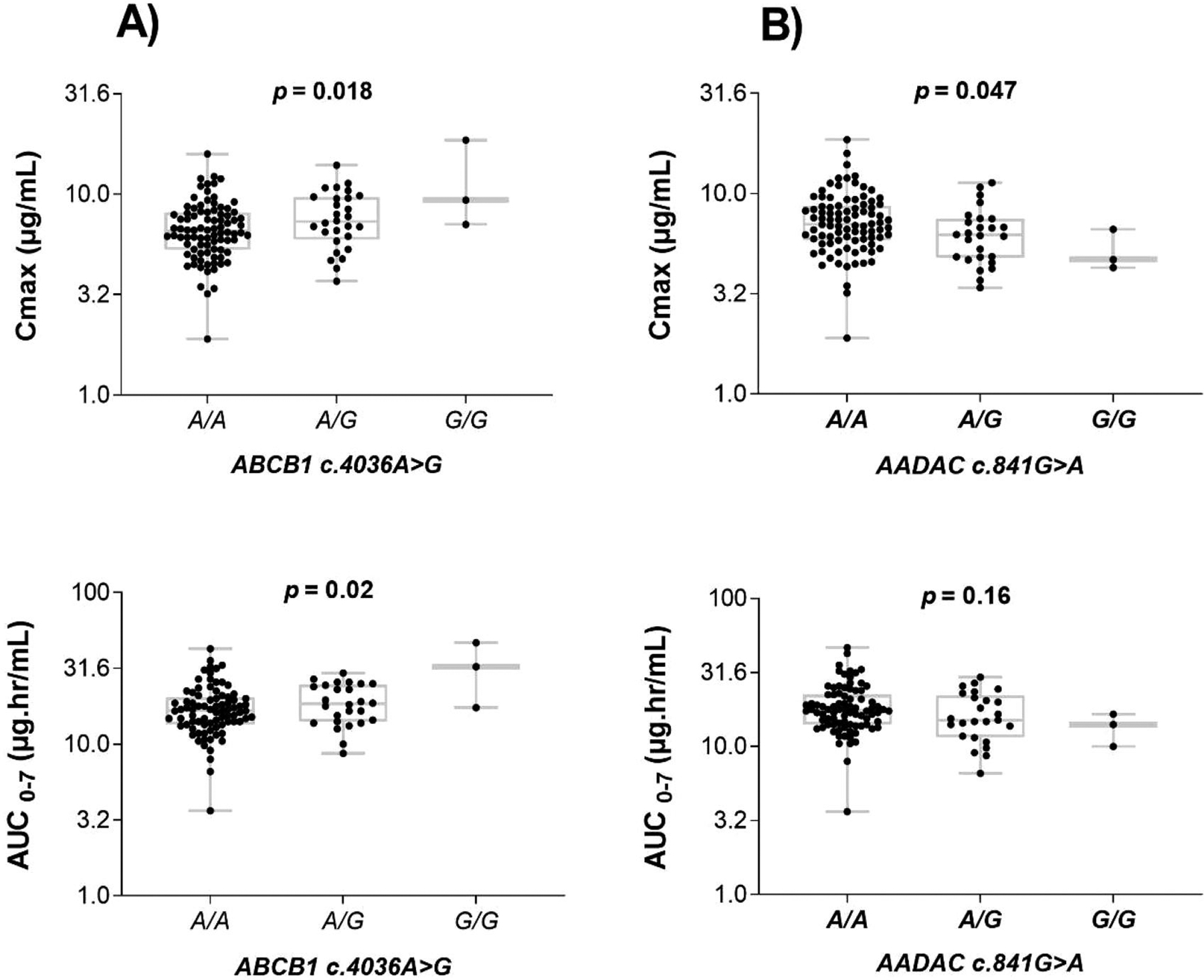
Comparison of rifampicin *C*_max_ and AUC_0–7 h_ in the *ABCB1 c.4036A>G* (right) and *AADAC2 c.841G>A* (left) genotypes. The box plots show the median ± interquartile range, whereas whiskers denote the minimum and maximum values.

Significant variability in rifampicin *C*_max_ (*p* = 0.018) and AUC_0–7 h_ (0.02) between the *ABCB1 c.4036A>G* genotype groups was observed. The geometric mean of *C*_max_ and AUC_0–7 h_ was significantly higher among patients homozygous for the variant allele *ABCB1c.4036G/G* than heterozygous A/G or homozygous wild type (A/A)(Table 3). A further post hoc analysis using Bonferroni correction indicated significant differences in *C*_max_ (*p* = 0.036) and AUC_0–7 h_ (*p* = 0.023) between homozygous *ABCB1 c.4036 A/A* and homozygous wild-type (*G/G*) groups. The comparison of *C*_max_ and AUC_0–7 h_ between the different *ABCB1 c.4036A>G* genotype groups is presented in Figure 1(A). No significant difference in *C*_max_ and AUC_0–7_ was observed in the different *ABCB1 c.3435C>T* genotype groups.

Furthermore, a significant association of *AADAC c.841G>A* genotype with rifampicin *C*_max_ (*p* = 0.047) and a similar trend for AUC_0–7_ (*p* = 0.16) was observed and was lower in the wild type (*G/G*) genotype than heterozygous (*A/G*) or homozygous for *A* variant allele (*A/A*) (Figure 1(B)). However, a post hoc test showed no significant variation for AUC_0–7_ among the pairs of all three genotypes of *AADAC c.841G>A*. There was no significant association of *CES 2 c.269-965A>G* genotype with rifampicin *C*_max_ and AUC_0–7 h_.

### Predictors of rifampicin pharmacokinetics

A univariate followed by a multivariate analysis was conducted to identify predictors of *C*_max_ and AUC_0–7h_ using log10 transformed concertation data. Table 4 shows the results of univariate and multivariate analyses of associations between variables and rifampicin *C*_max_ and AUC_0–7 h_. In univariate analysis, *ABCB1 c.4036A>G*, *AADAC c.841G>A* genotypes and rifampicin dose were significant predictors of rifampicin *C*_max_ (*p ≤* 0.05), and a nearly significant effect was observed for sex (*p* = 0.06). All variables with *p* value <0.2 were further tested in the multivariate regression model. In multivariate analysis, sex, rifampicin dose, *ABCB1 c.4036A>G* and *AADAC c.841G>A* genotypes remained independent predictors of rifampicin *C*_max_.

*ABCB1 c.4036A>G* and drug dose were significant predictors for rifampicin AUC_0–7 h_ in both univariate and multivariate analysis. In multivariate analysis, sex was also a predictor of rifampicin AUC_0–7 h_. Overall, females had higher exposure to rifampicin compared to males. Age, alcohol, cigarette and khat use, *SLCO1B1c.388A>G*, *SLCO1B1c.521T>C*, *ABCB1 c.3435C>T*, *CES-2 c.269-965A>G* genotypes and days on drug therapy did not predict rifampicin exposure (*C*_max_ and AUC_0–7 h_).

The stepwise multivariate regression analysis demonstrated that *ABCB1 c.4036A>G* genotypes independently accounted for 5.8% of the variability in rifampicin *C*_max_. Combining *AADAC c.841G>A* and *ABCB1 c.4036A>G* genotypes increased the explained variability to 10.8%. Additionally, 14% variability in rifampicin *C*_max_ was observed when the drug dose (mg) was added to the two genotypes. The overall variability in rifampicin *C*_max_ explained by the two genotypes, drug dose and sex was 17.2%. Similarly, *ABCB1 c.4036A>G* genotypes explained 6.1% of the variability in rifampicin AUC_0–7_ explained by. With the sequential addition of sex, drug dose and *AADAC c.841G>A* to the model, the variability in rifampicin AUC_0–7_ increased to 10.1%, 15.8%, and 19.3%, respectively. These findings underscore the significant roles of *AADAC c.841G>A* and *ABCB1 c.4036A>G* genotype, along with sex and drug dose in predicting rifampicin *C*_max_ and AUC_0–7_ among the variables examined.

## Discussion

The study is the first to examine the relationship between genetic polymorphism and rifampicin pharmacokinetics in the Ethiopian population. We investigated the between-patient variability of rifampicin pharmacokinetics parameters (*C*_max_ and AUC_0–7 h_) in Ethiopian adults commencing TB treatment and the role of pharmacogenetic variations in drug transporter proteins (*SLCO1B1* and *ABCB1*) and metabolising enzymes relevant for rifampicin disposition (*AADAC2* and *CES2*). There were several notable findings. First, there was substantial between-patient variability in rifampicin plasma concentrations. Second, a majority (70%) of patients had rifampicin plasma concentrations below the recommended target (≥8 μg/mL). Third, rifampicin dose, *ABCB1c.4036A>G* and *AADACc.841G>A* genotypes and to some extent, sex were independent predictors of rifampicin *C*_max_ and AUC_0–7 h_.

Two weeks after treatment initiation, a 2-h post-dose plasma sample is recommended for therapeutic drug monitoring to predict TB treatment outcomes. Rifampicin *C*_max_ should exceed 8 mg/L for optimal therapeutic efficacy [32–34]. This peak concentration was not attained in about 70% of our patients who received the standard rifampicin dose. Our finding is in line with previous studies reporting that many patients receiving first-line anti-TB therapy do not achieve the rifampicin *C*_max_ target concentration, but the proportion varies between populations [16,34–36]. To the best of our knowledge, the proportion of TB patients below the target 8 mg/mL in this study is one of the highest. This finding is of concern since subtherapeutic levels are associated with unfavorable outcomes and risk for development of drug resistance [9,37]. Indeed, drug-resistant TB is an increasing concern in Ethiopia [5,38,39]. A higher dose of rifampicin or therapeutic drug monitoring in selected patients could be beneficial as suggested previously [9,40]. Whether high doses of rifampicin are safe and more effective than the standard dose is studied in clinical trials to shorten treatment duration and increase efficacy. The trial results indicated that a higher dose of rifampicin led to faster sputum sterilisation while maintaining a comparable level of toxicity to the standard dose [41–44]. Therefore, an increase in the dose of rifampicin in Ethiopian population may be warranted.

Several factors could contribute to the observed low rifampicin plasma concentrations in Ethiopian patients including genetic variations, malnutrition and HIV infection, which are quite prevalent in East Africa including Ethiopia [1,45,46]. However, compared to the 70% observed in this study, only 35% of Tanzanian TB patients had a rifampicin *C*_max_ below 8mg/L [47]. The low rifampicin concentrations in Ethiopian TB patients could be due to either higher rifampicin metabolising enzyme activities or increased autoinduction due to pharmacogenetic variations [23,25,26,48,49]. Lower plasma drug concentrations have been reported in earlier studies of antiretrovirals due to higher drug-metabolising enzyme activity and unique pharmacogenetic variation in Ethiopians compared to other populations, including Tanzanians [23,25,26,50]. Our study highlights the existence of substantial differences in rifampicin pharmacokinetics between populations in sub-Saharan Africa and findings from one population may not be directly extrapolated to others on the continent. Recently we reported high plasma isoniazid concentrations and a high prevalence of slow N-acetyltransferase 2 (NAT2) acetylators in Ethiopian TB patients [51].

There have been inconsistent results about the effects of *SLCO1B1* genetic variation on rifampicin exposure. Previous studies in South African and Ugandan patients reported an association of the *SLCO1B1* genotype with variability in rifampicin pharmacokinetics [21,22,52]. However, this finding was not replicated in many studies [11,15,53,54]. Likewise, we found no significant impact of *SLCO1B1 c.388A>G* and *SLCO1B1 c.521T>C* on rifampicin *C*_max_ and AUC_0–7h_. SLCO1B1*1B and SLCO1B1*5 are missense mutations, involving the change of asparagine to aspartic acid at position130 and valine to alanine at position 174, respectively (Table 2). The variant alleles SLCO1B1*1B and SLCO1B1*5 were associated with increased and decreased transporter activity of OATP1B1, respectively. SLCO1B1*1B, which is associated with higher transporter activity, occurs at a higher frequency (62.2%) in Ethiopians and Tanzanians (86.8%) than in Europeans (34.2%) [23]. On the other hand, the defective *SLCO1B1c.521T>C* variant allele caused reduced enzyme activity occurs at a lower frequency among Ethiopians (2.8%) than Tanzanians (4.7%) or Europeans (8%) [23,25,26].

Rifampicin is a substrate and inducer of P-gp which is a product of the *ABCB1* gene [40,55]. Few studies have evaluated the effect of *ABCB1* gene polymorphism on rifampicin pharmacokinetics. Huerta-García et al. reported that patients with *CC* or *CT* genotypes of *ABCB1* (*c.3435C>T*) had lower *C*_max_ and AUC_24_ than those with a *TT* genotype [56]. The *TT* homozygous genotype had significantly lower P-gp expression in the small intestine and showed the highest plasma concentrations of some drugs after oral administration [24]. However, we found no significant variation in rifampicin *C*_max_ and AUC_0–7 h_ for *ABCB1 c.3435C>T*. The *ABCB1c.4036A>G* genotype, which is in linkage disequilibrium with *c.3435C>T,* significantly influenced between-patient variability of rifampicin *C*_max_ and AUC_0–7 h_. Rifampicin AUC_0–7 h_ was significantly higher in homozygous variant genotype (*GG*) carriers compared to the homozygous wild-type A/A (Figure 1). Nevertheless, the homozygous variant genotype (*GG*) occurs at a low frequency in our study population, consistent with findings from a previous report [49].

Few studies have investigated the impact of *AADAC* and *CES* genetic polymorphism on rifampicin pharmacokinetics. The association of *CES-2 c.−2263A>G* (rs3759994) in the promotor region and closely linked to *c.269-965A>G* (rs4783745) and c.1612 + *136 G>A* with increased rifampicin exposure is reported [13]. Patients who carry the *CES2* (rs8192925) *G* versus *A* allele had a 17.2% increase in rifapentine AUC_0–24_ (14). In our study, there was no significant association of *CES2 c.269-965A>G* genotypes with rifampicin *C*_max_ and AUC_0–7 h_. Likewise, no significant effect of *CES-2* on rifampicin exposure variability was observed in Ghanaian children [16]. *AADAC* and *CES-1* genotypes were not associated with rifampicin pharmacokinetics in Malawian TB patients [15].

We found a significant association between *AADAC c.841G>A* genotype and rifampicin *C*_max_, which was significantly higher in carriers of the mutant variant allele (A/A, G/A) than in those with wild-type G/G genotype (Figure 1). Our result is consistent with previous reports [3,14]. Francis et al. reported that patients with *A/A* genotype had a lower rifapentine clearance. Similarly, a previous study found an association of *AADAC c.841G* variant allele with low rifapentine AUC, particularly in black patients [14]. However, this finding was not observed in Malawian adult TB patients [15]. The low frequency of a wild-type (*GG*) genotype in Malawians may have contributed to the differing results. Indeed, the frequency distribution of AADAC*2 (c.841G>A) exhibits considerable variability across races and populations. Notably, the reported allele frequencies of AADAC*2 among European American, African American, Japanese and Korean populations were around 60%, contrasting with the 99.9% prevalence in Peruvian TB patients [57] where the wild-type variant is almost missing. Our study among Ethiopian TB patients reveals AADAC*2 allele frequencies of 86%, and the wild-type *G* variant was less prevalent with only three individuals exhibiting homozygosity for *G/G* genotype. This underscores the need for further investigation in populations where the *AADAC c.841G* variant occurs at higher frequencies to replicate and confirm our findings.

In addition to genetic polymorphism, other predictors such as age, sex, duration of therapy with rifampicin, drug dose and substance use were tested in univariate followed by multivariate analyses. Sex and drug dose were significantly associated with rifampicin *C*_max_ and AUC_0–7 h_ in multivariate analysis. Females had higher rifampicin exposure (higher *C*_max_ and AUC_0–7 h_) than males. This is consistent with previous studies where male sex was associated with lower rifampicin exposure [35,36,52,58].

Our study presents the first insight into the extent of variability in rifampicin exposure (*C*_max_ and AUC_0–7_) and the impact of genetic variation in drug transporters and metabolising enzymes in Ethiopian TB patients. However, it is imperative to acknowledge certain limitations in our study. The estimation of rifampicin pharmacokinetics in our study relied on three sampling time points within 7 h post-dose, adhering to the recommended approach for therapeutic drug monitoring [31]. A 2-h post-dose sample approximates the *C*_max_ for most TB drugs and adding a 6-h sample allows the clinician to distinguish between delayed absorption and malabsorption [31,32,34]. Nevertheless, although the spare sampling strategy is useful for capturing the AUC_0–24 h_ [59], the three time point concentration dataset in our study may not entirely capture the AUC accurately. Nevertheless, it is crucial to underscore that obtaining multiple blood samples solely for the study’s objectives from newly diagnosed TB-infected patients undergoing an intensive phase of treatment is impractical and raises ethical concerns.

Furthermore, in our study population, the occurrence of the wild-type *AADAC c.841 G/G* and the variant *ABCB1 c.4036 G/G* genotype occurred at a lower frequency, potentially influencing the association of rifampicin *C*_max_ and AUC_0–7 h_ with the investigated genotypes. It is note-worthy that globally, and particularly within Africa, G variant alleles exhibit lower frequencies for both *AADAC c.841G>A* and *ABCB1 c.4036A>G*. The frequency of *ABCB1 c.4036A>G* varies among black Africans, ranging from 29% in Tanzanians [60] to 18% in Ethiopians [28]. Considering these variations, future large-sample studies across diverse populations in high TB-burden areas, including Africa, where rifampicin is a cornerstone of TB therapy, are recommended to validate and replicate our findings.

In conclusion, we report low rifampicin exposure and high variability in rifampicin *C*_max_ and AUC_0–7_ in about two-thirds of Ethiopian TB patients. Rifampicin exposure varied with sex, dose, *ABCB1 c.4036A>G* and *ADAC c.841G>A* genotypes. *AADAC c.841GG* and *ABCB1 c.4036A>GAA* genotype groups and male patients had a higher risk of low rifampicin plasma exposure than females. *SLCO1B1 c.388A>*, *SLCO1B1 c.521T>C*, *ABCB1 c.3435C>T* and *CES2 c.269-965A>G* genotypes did not affect rifampicin exposure. The impact of low rifampicin exposure on treatment outcomes needs further investigation in Ethiopian TB patients. Our findings may have important clinical implications and warrant studies on whether high-dose rifampicin improves therapeutic efficacy.

## Figures and Tables

**Table 1.** Sociodemographic and clinical characteristics of 119 Ethiopian tuberculosis patients.

Variables		Pulmonary TB (*n* = 78)	Extrapulmonary TB (*n* = 41)	All patients (*n* = 119)
Sex (*n*)	Male	45	17	62 (52.5%)
	Female	33	24	57 (47.5%)
Smoking (*n*)	Yes	15	1	16 (13.4%)
	No	63	40	103 (86.6%)
Khat chewer (*n*)	Yes	20	2	22 (18.5%)
	No	58	39	97 (81.5%)
Alcohol (*n*)	Yes	17	3	20 (16.8%)
	No	61	38	99 (83.2%)
Age (years), median (IQR)		26 (21–35)	28 (24.5–36)	28 (22–35)
Median body weight in kg (IQR)		53 (45–60)	58 (52.5–68.5)	54.75 (48–61.75)
Drug dose (mg/kg, *SD*)		9.46 (0.99)	9.26 (0.98)	9.39 (0.98)
Median *C*_max_, μg/mL (IQR)		6.45 (5.13–8.54)	7.46 (6.02–8.72)	6.75 (5.39–8.58)
Median AUC_0–7_ μg.h/mL (IQR)		16.52 (13.81–21.98)	17.55 (14.3–22.59)	17.05 (13.87–22.26)

AUC_0–7 h_: area under the time-concentration curve; *C*_max_: maximum concentration; *n*: number; IQR: interquartile range; *SD*: standard deviation.

**Table 2.** Genotype and variant allele frequency of *SLCO1B1*, *ABCB1*, *AADAC* and *CES-2*.

Variant allele	Protein	Genotype frequency (*n*, %)			Allele frequency (%)		χ^2^	*p* value
SLCO1B1*1B (*c.388A>G*)	Asn130Asp	*A/A* (15, 12.6)	*A/G* (60, 50.4)	*G/G* (37, 44)	*A* (37.7)	*G* (62.2)	0.618	0.43
SLCO1B1*5 (*c.521T>C*)	Val174Ala	*T/T* (76, 63.9)	*T/C* (38, 31.9)	*C/C* (5, 4.2)	*T* (79.8)	*C* (20.2)	0.008	0.99
*ABCB1 c.3435C>T*	Ile1145Ile	*C/C* (67, 56.3)	*C/T* (46, 38.7)	*T/T* (6, 5.0)	*C* (75.6)	*T* (24.4)	0.28	0.59
*ABCB1c.4036A>G*	Located in 3′-UTR	*A/A* (88, 73.8)	A/G (28, 23.5)	*G/G* (3, 2.5)	*A* (85.4)	*G* (14.6)	0.183	0.67
AADAC*2 (*c.841G>A*)	Val281Ile	*G/G* (3, 2.5)	*G/A* (26, 21.7)	*A/A* (90, 75)	*G* (13.9)	*A* (86.1)	0.447	0.5
*CES-2 c.269-965A>G*	Located in intron 1	*A/A* (55, 46.2)	*A/G* (55, 46.2)	*G/G* (9, 7.6)	*A* (69.4)	*G* (30.6)	0.896	0.34

*n*: number; UTR: untranslated region. The chi-square test and *p* value show correlations between the observed and expected genotype frequencies according to the Hardy–Weinberg equilibrium.

**Table 3.** Effects of *SLCO1B1*, *ABCB1*, *AADAC* and *CES-2* genotype on rifampicin *C*_max_ and AUC_0–7 h_ in Ethiopian TB patients (*n* = 119).

Genotype		*N*	*C*_max_ (μg/mL)			AUC_0–7_ (μg h/mL)		
			Median (IQR)	Geometric mean ± *SE*	*p* Value*	Median (IQR)	Geometric mean ± *SE*	*p* value*
SLCO1B1*1B (c.388A>G)	A/A	15	6.88 (5.83–9.36)	7.08 ± 1.1	0.87	17.95 (16.59–20.93)	17.78 ± 1.12	0.67
	A/G	60	6.62 (6.23–7.75)	6.76 ± 1.05		16.35 (14.36–18.22)	16.6 ± 1.05	
	G/G	44	6.82 (6.1–7.4)	6.92 ± 1.05		17.14 (15.35–18.78)	17.78 ± 1.07	
SLCO1B1*5 (c.521T>C)	T/T	76	6.59 (6.18–7.02)	6.76 ± .05	0.15	16.65 (15.08–18.12)	16.98 ± 1.05	0.18
	T/C	38	7.62 (6.76–8.12)	7.24 ± 1.07		18.49 (17.03–22.4)	18.2 ± 1.07	
	C/C	5	5.1 (4.76–7.41)	5.37 ± 1.1		12.5 (12.44–19.61)	13.18 ± 1.12	
ABCB1 c.3435C>T	C/C	67	6.42 (6.1–7.2)	6.61 ± 1.05	0.70	16.7 (15.08–18.22)	16.98 ± 1.05	0.87
	C/T	46	7.23 (6.63–7.95)	6.92 ± 1.07		17.85 (16.48–19.73)	17.78 ± 1.07	
	T/T	6	6.76 (6.1–10.43)	7.24 ± 1.15		14.68 (13.4–23.99)	16.6 ± 1.12	
ABCB1 c.4036A>G	A/A	88	6.53 (6.10–7.18)	6.61 ± 1.05	0.018	16.7 (15.08–17.77)	16.6 ± 1.05	0.02
	A/G	28	7.29 (6.59–8.85)	7.41 ± 1.07		18.31 (15.5–23.09)	18.2 ± 1.07	
	G/G	3	9.35 (7.07–18.57)	10.72 ± 1.32		32.13 (17.3–47.05)	29.51 ± 1.35	
AADAC2 c.841G>A	A/A	90	7.045 (6.49–7.82)	7.08 ± 1.05	0.047	17.56 (16.48–18.58)	17.78 ± 1.05	0.16
	G/A	26	6.21 (4.85–6.79)	6.03 ± 1.07		15.06 (14.11–20.29)	15.49 ± 1.07	
	G/G	3	4.69 (4.27–6.63)	5.13 ± 1.15		13.99 (9.92–16.59)	13.18 ± 1.17	
CES-2 c.269-965A>G	A/A	55	7.18 (6.59–8.04)	7.08 ± 1.05	0.08	17.85 (15.5–19.06)	17.38 ± 1.05	0.19
	A/G	55	6.42 (6.10–7.2)	6.31 ± 1.05		16.58 (14.82–17.3)	16.22 ± 1.05	
	G/G	9	6.87 (6.18–13.94)	8.13 ± 1.15		18.32 (15.08–29.74)	21.38 ± 1.15	

AUC_0–7 h_: area under the time-concentration curve; *C*_max_: maximum concentration; *n*: number; IQR: interquartile range; *SE*: standard error; GM: geometric mean; TB: tuberculosis.

* *p* value from analysis of variances using log_10_ transformed *C*_max_ and AUC_0–7 h_ data.

**Table 4.** Univariate and multivariate linear regression analysis of factors associated with rifampicin log_10_*C*_max_ and log_10_AUC_0–7 h_ in Ethiopian adult tuberculosis patients.

Variable	*C*_*max*_				AUC			
	Univariate		Multivariate		Univariate		Multivariate	
	Beta coefficients (95% CI)	*p* value	Adjusted beta coefficients (95% CI)	*p* value	Beta coefficients (95% CI)	*p* value	Adjusted beta coefficients (95% CI)	*p* value
Age	0.002 (−0.001 to 0.006)	0.12	0.002 (−0.048 to 0.004)	0.24	0.002 (−0.001 to 0.006)	0.19	0.002 (−0.002 to 0.005)	0.31
Sex (female vs. male)	−0.051 (−0.11 to 0.003)	0.06	−0.056 (−0.11 to 0.004)	0.03	−0.057 (−0.12 to 0.04)	0.07	−0.063 (−0.12 to 0.03)	0.04
Drug dose (mg)	0.00 (0.00 to 0.001)	0.05	0.000 (0.00 to 0.001)	0.03	0.000 (0.00 to 0.01)	0.05	0.000 (0.00 to 0.01)	0.03
Alcohol use (no vs. yes)	0.00 (−0.066 to 0.8)	0.84			0.002 (−0.08 to 0.083)	0.97		
Khat chewing (no vs. yes)	0.00 (−0.074 to 0.067)	0.91			0.013 (−0.066 to 0.093)	0.74		
Smoking (no vs. yes)	−0.012 (−0.093 to 0.067)	0.75			0.016 (−0.076 to 0.11)	0.73		
Days on drug therapy	−0.001 (−0.004 to 0.0012)	0.43			0.001 (−0.005 to 0.003)	0.59		
*SLCO1B1c.388A>G*	−001 (−0.043 to 0.04)	0.96			0.007 (−0.04 to 0.054)	0.76		
*SLCO1B1c.521T>C*	0.002 (−0.05 to 0.046)	0.94			0.002 (−0.052 to 0.056)	0.95		
*ABCB1 c.3435C>T*	0.02 (−0.026 to 0.066)	0.4			0.006 (−0.046 to 0.059)	0.81		
*ABCB1 c.4036A>G*	0.071 (0.018 to 0.124)	0.009	0.063 (0.013 to 0.114)	0.015	0.071 (0.011 to 0.13)	0.02	0.059 (0.001 to 0.13)	0.048
AADAC*2c.841G>A	−0.068 (−0.122 to −0.014)	0.01	−0.065 (−0.12 to −0.013)	0.015	−0.059 (−0.12 to 0.001)	0.06	−0.059 (−0.12 to 0.001)	0.053
*CES-2 c.269-965A>G*	−0.004 (−0.048 to0.04)	0.86			0.008 (−0.043 to0.058)	0.76		

AUC_0–7 h_: area under the time-concentration curve; *C*_max_: maximum concentration; CI: confidence interval.
